# CD14 is critical for TLR2-mediated M1 macrophage activation triggered by N-glycan recognition

**DOI:** 10.1038/s41598-017-07397-0

**Published:** 2017-08-01

**Authors:** Thiago Aparecido da Silva, André L. V. Zorzetto-Fernandes, Nerry T. Cecílio, Aline Sardinha-Silva, Fabrício Freitas Fernandes, Maria Cristina Roque-Barreira

**Affiliations:** Departamento de Biologia Celular e Molecular e Bioagentes Patogênicos, Faculdade de Medicina de Ribeirão Preto-USP, Ribeirão Preto, 14049-900 São Paulo Brazil

## Abstract

Agonist interaction with Toll-like receptors (TLRs) induces T cell-mediated immunity, which is effective against intracellular pathogens. Consequently, TLR agonists are being tried as immunomodulatory agents. The lectin ArtinM targets TLR2 N-glycans on macrophages, induces cytokines production, and promotes T helper-1 immunity, a process that culminates in resistance to several parasitic and fungal infections *in vivo*. Because co-receptors influence agonist binding to TLRs, we investigated whether CD14 is required for macrophage activation induced by ArtinM. Macrophages from wild-type mice stimulated by ArtinM not only produced cytokines but also had the following activation profile: (i) expression of M1 polarization markers; (ii) nitrite oxide production; (iii) cellular migration; (iv) enhanced phagocytic and fungicide activity; (v) modulation of TLR2 expression; and (vi) activation of NF-κB pathway. This activation profile induced by ArtinM was evaluated in macrophages lacking CD14 that showed none of the ArtinM effects. We demonstrated by immunoprecipitation and sugar inhibition assays the physical interaction of ArtinM, TLR2, and CD14, which depends on recognition of the trimannoside that constitutes the core of N-glycans. Thus, our study showed that CD14 is critical for ArtinM-induced macrophage activation, providing fundamental insight into the design of anti-infective therapies based on carbohydrate recognition.

## Introduction

The antibodies produced after vaccination neutralize the inoculums of extracellular pathogens and prevent infections^1^. However, this effective formula is not applied to prevent infections with intracellular pathogens^2^ because T-cell-mediated immunity is required to eliminate these pathogens. Recent studies demonstrate that Toll-like receptor (TLR) agonists may promote protection against infections by intracellular pathogens^3–6^. Indeed, the interaction of TLRs with their respective ligands promotes conformational changes that cause the cytoplasmic domains of these receptors to associate with intracellular sorting adaptor proteins^7, 8^. These molecules, in turn, induce the recruitment of signaling adaptors MyD88 and TIR domain-containing adaptor-inducing interferon-β (TRIF), with the resulting formation of supramolecular organizing centers^9^. Then, mitogen activated kinases (MAPKs) and the nuclear factor-κB (NF-κB), are activated and engage downstream transcription factors, resulting in the production of inflammatory and anti-inflammatory mediators^10^. These mediators, beyond providing early mechanisms of host defense, influence the antigen-specific adaptive response^11^. Indeed, the stimulation of TLR2 on antigen presenting cells (APC) favors Th1 immunity^12–15^. Advantageously, TLR2-mediated activation also contributes to the maintenance of Th1 response^16^.

TLR2, unlike other TLRs, forms heterodimers with TLR1 or TLR6 to recognize microbe components such as lipoteichoic acids (LTA), peptidoglycan (PGN), and lipopeptides (LP)^17^. Agonist-TLR2 interaction is influenced by co-receptors such as the cluster of differentiation 14 (CD14), CD36, and integrins^17^. CD14, a glycosylphosphatidylinositol-anchored glycoprotein expressed primarily on the surface of myeloid cells, has a solenoid-shaped structure with three sites for N-glycosylation that are occupied by oligosaccharides essential for protein secretion and proper functioning^18, 19^. Although characterized as a co-receptor for the TLR4 response to lipopolysaccharide (LPS), CD14 also participates in the activation of several extracellular and endosomal TLRs^17^. In this context, CD14 binding to triacylated lipopeptides enhances recognition by the TLR2/1 complex, which favors TLR2 heterodimerization and lipid raft recruitment^20^.

Because TLR activation directs the adaptive immunity^21^, TLR agonists have been used as adjuvants for treating human diseases^22^. Trials of new drugs based on natural or synthetic TLR ligands to combat human infectious diseases are ongoing^23^. We previously reported that ArtinM, a D-mannose-binding lectin^24^, confers protection against intracellular parasites and fungal infections^25–30^. This activity was attributed to ArtinM interaction with TLR2 N-glycans^31^, which is followed by IL-12 production and establishment of Th1 immunity. The ArtinM effects were shown to depend on carbohydrate recognition because direct binding to TLR2 was inhibited by the trimannoside specifically targeted by ArtinM^32, 33^, which constitutes the core of N-glycans. These data demonstrated that a carbohydrate recognition protein might function as a TLR agonist and exert immunomodulatory activity. We also reported that lectins derived from *Paracoccidioides brasiliensis* ^34, 35^ and *Toxoplasma gondii* ^36^ interact with N-glycans of both TLR2 and TLR4 and confer protection against the corresponding infections.

Considering the role of CD14 in TLR2 activation, we investigated the involvement of CD14 in macrophage activation triggered by the TLR2 agonist ArtinM. We found that CD14 is crucial for the activities exerted by ArtinM on macrophages because its absence resulted in blockages of ArtinM-induced cytokine production, M1 polarization, cellular migration, and phagocytic properties. These processes are associated with the inhibition of NF-κB. An immunoprecipitation assay showed that the interaction of ArtinM, TLR2, and CD14 is synergistic and inhibited by the presence of the ArtinM-specific trimannoside. Elucidating the details of ArtinM interaction with CD14 allows a better understanding of the structural basis of immunomodulation induced by carbohydrate recognition and provides fundamental insights for the design of lectin-mimetic drugs^37^ for anti-infective therapies.

## Results

### Requirement of CD14 for the production of pro-inflammatory mediators by ArtinM-stimulated macrophages

**Figure 1:**
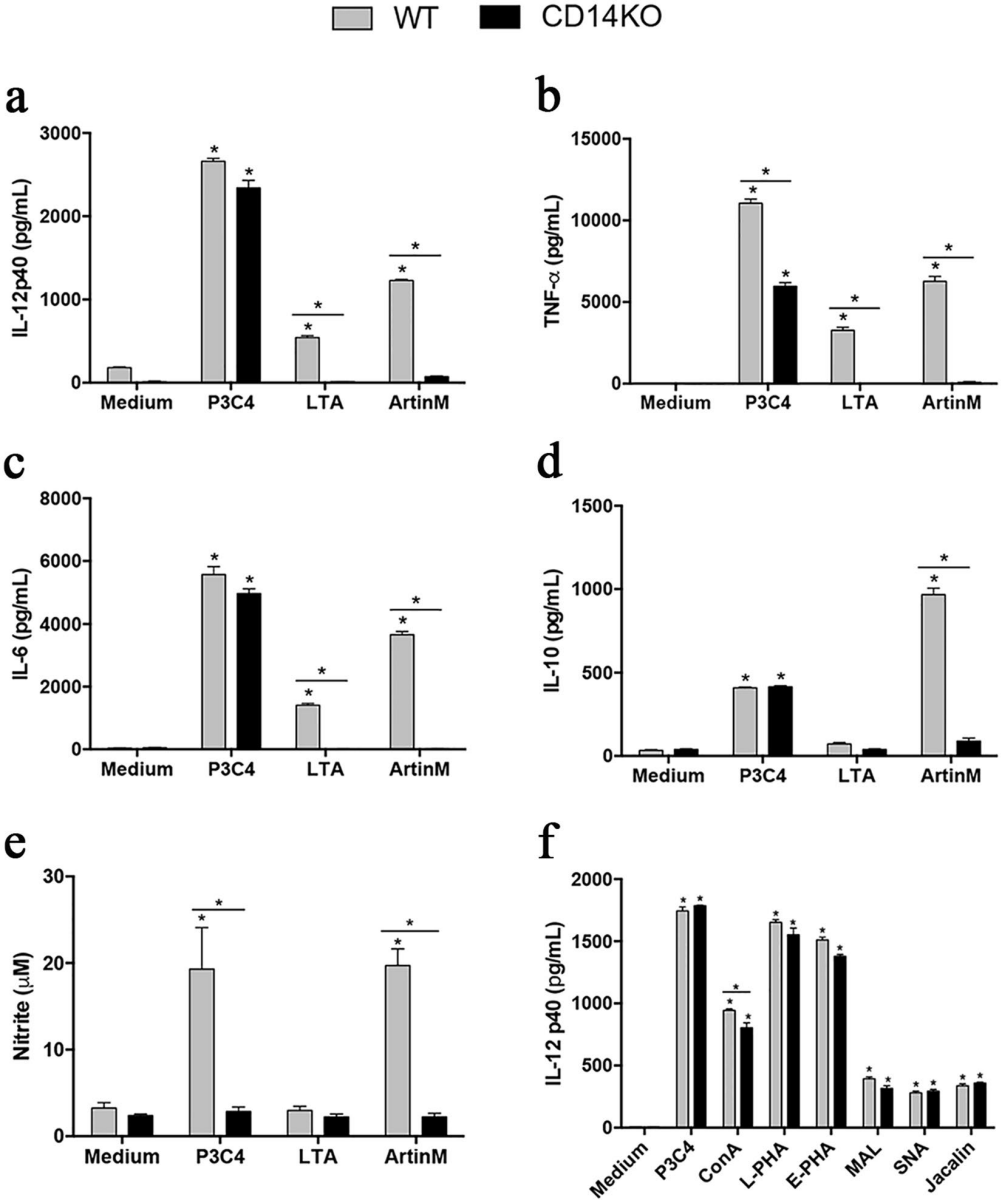
Influence of the absence of the CD14 in pro-inflammatory mediators production by ArtinM-stimulated macrophages. Macrophages (2 × 10^6^/mL) from wild-type (WT) and CD14 knockout (KO) mice were stimulated with ArtinM (2.5 µg/mL), Concanavalin A (ConA; 10 µg/mL), Phytohemagglutinin-L (L-PHA; 10 µg/mL), Phytohemagglutinin-E (E-PHA; 10 µg/mL), Maackia amurensis lectin (MAL; 10 µg/mL), Sambucus nigra lectin (SNA; 10 µg/mL), Jacalin (10 µg/mL), palmitoyl-3-cysteine-serine-lysine-4 (P3C4; 1 µg/mL), lipoteichoic acid (LTA; 1 µg/mL) or medium for 48 h. Culture supernatants were assessed by ELISA for levels of IL-12p40 (**a** and **f**), TNF-α (**b**), IL-6 (**c**), IL-10 (**d**), and nitrite (**e**). The values (in pg/mL or µM) in WT and CD14 KO macrophages were compared with those of cells in medium; results provided by WT and CD14 KO macrophages stimulated with ArtinM, P3C4, or LTA were also compared. Data are shown as means ± SEM of independent experiments. *p < 0.05; one-way analysis of variance followed by Bonferroni’s test.

Interest in modulating immunity has motivated the search for various TLR ligands with possible pharmaceutical applications. Among these, plant lectins are represented by ArtinM, which interacts with TLR2 N-glycans to induce cell activation and pro-inflammatory cytokine production, culminating in the development of Th1 immunity. In this study, we determined whether the TLR co-receptor CD14 influences macrophage response to ArtinM. To this end, peritoneal macrophages obtained from C57BL/6 (wild-type [WT]) or CD14 knockout (KO) mice were stimulated for 48 h with ArtinM, and the concentration of pro-inflammatory mediators was assessed in the culture supernatants. ArtinM induced a significant increase in the production of IL-12 p40, tumor necrosis factor-α (TNF-α), IL-6, IL-10, and nitric oxide (NO) by WT macrophages, as verified by the positive control, palmitoyl-3-cysteine-serine-lysine-4 -Pam3CSK4- (P3C4; Fig. 1a–e). In contrast, CD14KO macrophages did not enhance production of the inflammatory mediators in response to ArtinM, and behaved as unstimulated macrophages. Moreover, we investigated whether ArtinM at high concentration as high as 10 μg/mL could be able to induce IL-12 and NO production by CD14 KO macrophages. As shown in Fig. S1, the mediator’s production was persistently similar to that verified in the absence of a stimulus (medium). Regarding the positive controls, the stimulus with P3C4 induced IL-12p40, IL-6, and IL-10 production independently of CD14, whereas lipoteichoic acid (LTA) promoted IL-12p40, TNF-α, and IL-6 production in a manner entirely dependent on CD14 (Fig. 1). These findings reveal that the positive controls P3C4 and LTA diverge regarding CD14 dependence to induce the production of pro-inflammatory mediators. However, the requirement of CD14 for ArtinM activity was clearly demonstrated.

We then evaluated IL-12p40 production induced by six other plant lectins and determined whether the responses were dependent on CD14. Only three of the assayed lectins (Concanavalin A [ConA], Phytohemagglutinin-L [L-PHA], and Phytohemagglutinin-E [E-PHA]) are known to stimulate cytokine production in macrophages. Culture supernatants of the murine peritoneal macrophages of WT and CD14 KO mice stimulated with either lectins or P3C4 were assessed for IL-12p40 levels. L-PHA and E-PHA stimulated IL-12 production at levels closer to those stimulated by the positive control, P3C4. By contrast, lower but significant levels of IL-12p40 were induced by *Maackia amurensis* lectin (MAL), *Sambucus nigra* lectin (SNA), and Jacalin stimulation. The results of the same assay performed with CD14 KO macrophages showed that only ConA activity was impaired in the absence of CD14; however, none of the tested lectins showed the conspicuous dependence on CD14 of ArtinM. CD14 was not required for IL-12p40 production by macrophages stimulated by the remaining lectins (Fig. 1f). These findings indicate that CD14 is crucial for the ArtinM-induced production of IL-12 and, presumably, for the modulation of immunity. They also suggest that CD14 dependence pertains to ArtinM stimulation.

### CD14 accounts for the ArtinM-induced polarization of macrophages

**Figure 2:**
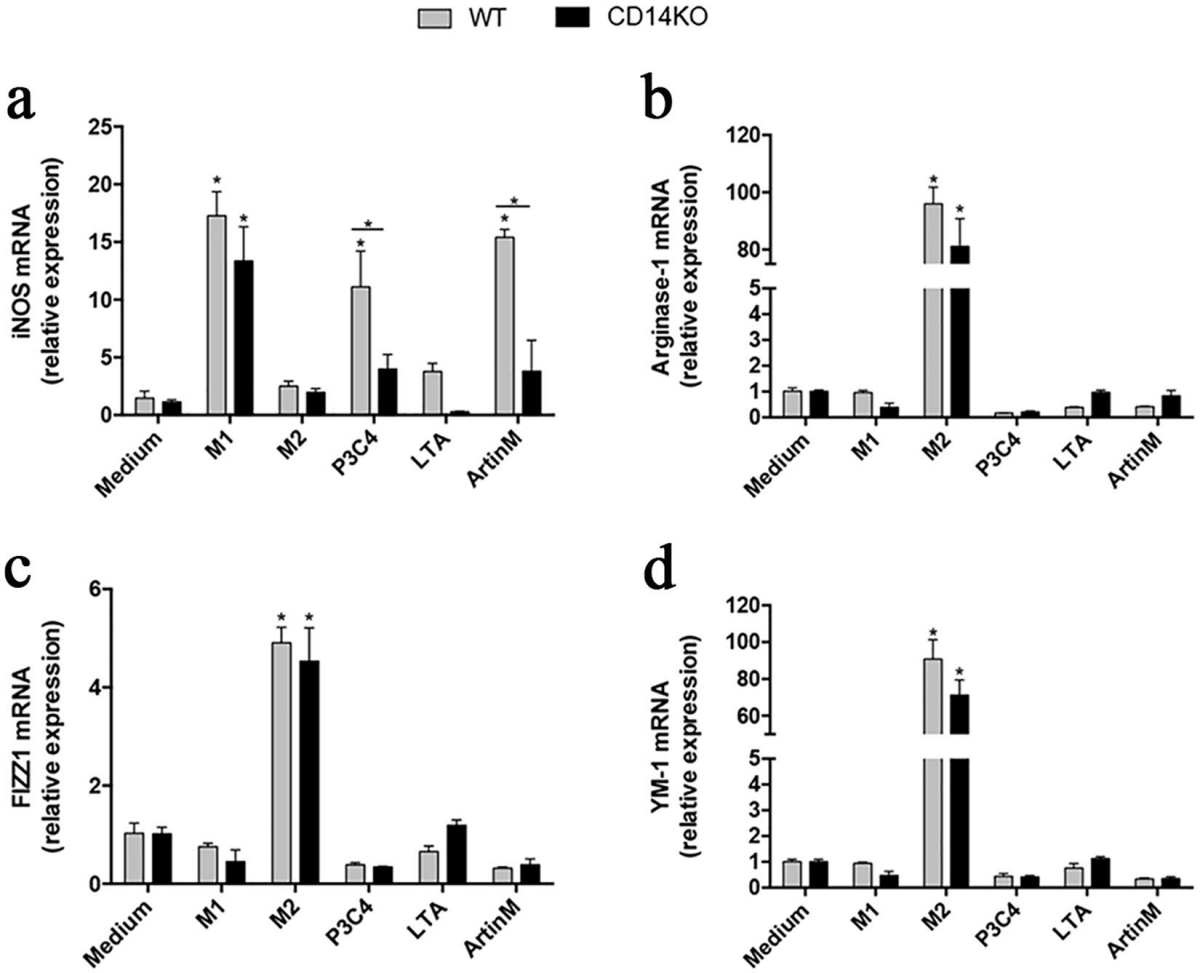
ArtinM activation of macrophages uses CD14 to promote M1 polarization. Macrophages (2 × 10^6^/mL) from the WT and CD14 KO mice were incubated with ArtinM (2.5 µg/mL), P3C4 (1 µg/mL), LTA (1 µg/mL), IFN-γ (M1; 50 ng/mL), IL-4 plus IL-10 (M2; 50 ng/mL), or medium for 10 h. The RNA samples extracted from these cells were reverse-transcribed into cDNA and analyzed for the expression of iNOS2, Fizz1, Arginase-1, and YM-1 by real-time polymerase chain reaction (PCR). The relative expression levels in WT and CD14 KO macrophages stimulated with M1, M2, P3C4, LTA, and ArtinM were compared with those of cells in medium, and the expression levels were also compared between WT and CD14 KO macrophages under similar conditions. Data are shown as means ± SEM of independent experiments. *p < 0.05, according to one-way analysis of variance and Bonferroni’s test.

After activation by certain stimuli, such as TLR ligands, macrophages may achieve functionally polarized states—namely M1 and M2—characterized by the expression of particular markers^38^. Considering that the activation of macrophages by ArtinM involves TLR2 and CD14, we investigated whether the lectin also induces macrophage polarization. Accordingly, we analyzed macrophages stimulated with ArtinM using real-time polymerase chain reaction (PCR) for the expression of traditional markers of M1 (inducible nitric oxide synthase 2 [iNOS2]) and M2 (Fizz1, Arginase-1 [Arg-1], and YM-1) polarization. We used IFN-γ and IL-4 plus IL-10 as inductors of M1 and M2, respectively. A 15-fold increase in iNOS2 expression by WT macrophages occurred in response to ArtinM but not to LTA (Fig. 2a). In CD14 KO macrophages, iNOS2 expression was abolished in response to ArtinM; this expression was also inhibited in response to P3C4 (Fig. 2a). In addition, ArtinM-stimulated WT macrophages increased STAT1 and IFN-β transcripts levels by 7- and 5-fold, respectively. Notably, CD14 KO macrophages were non-responsive to ArtinM, since the STAT1 and IFN-β transcripts levels were as low as that verified in the absence of stimulus (i.e. medium only) (Fig. S2).

Regarding M2 markers, ArtinM stimulation of WT and CD14KO macrophages did not alter the Fizz1, arginase-1 (Arg-1), or YM-1 expression levels, which were comparable to those detected in unstimulated WT and CD14KO macrophages (Fig. 2b–d). Stimulation with P3C4, LTA, and IFN-γ (M1) had no effect on M2 markers in either WT or CD14KO macrophages, but M2 stimuli (IL-4 plus IL-10) induced a significant increase in the levels of Fizz1, Arg-1, and YM-1 transcripts (Fig. 2b–d). These results indicate that ArtinM induces M1 polarization of macrophages through a mechanism that is crucially dependent on CD14.

### CD14 is required for the enhancement of the effector functions of ArtinM-stimulated macrophages

**Figure 3:**
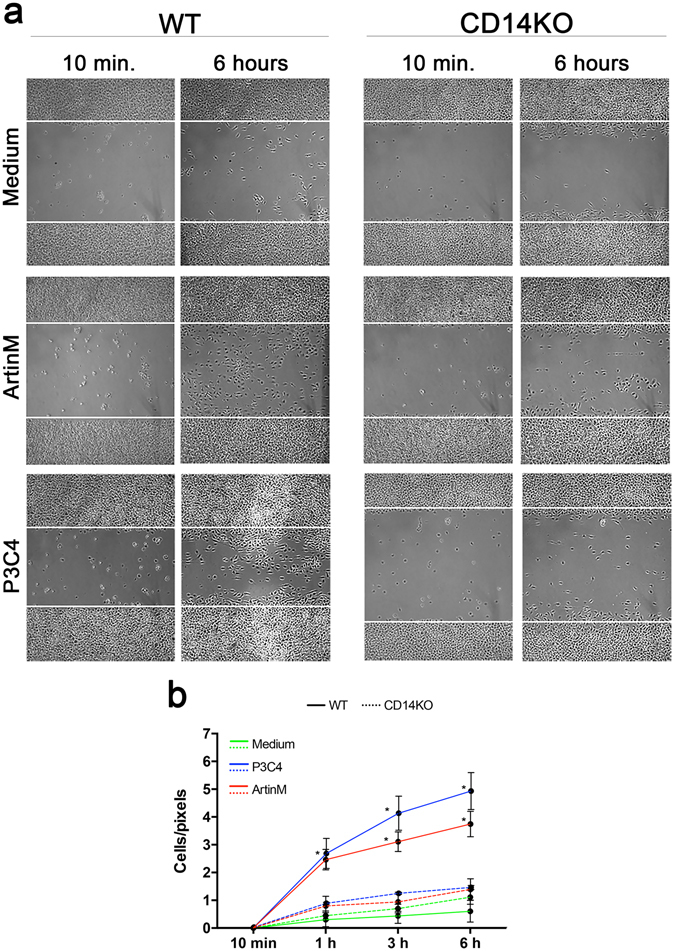
Macrophage migration induced by ArtinM is inhibited in the absence of CD14. Macrophages (1 × 10^6^/well) from WT and CD14 KO mice were assayed for migration in a wound healing model. (**a**) Cell monolayers were scratched (bounded by white line) and stimulated with ArtinM (2.5 µg/mL), P3C4 (1 µg/mL), or medium. Images of the scratch wounds were acquired at 10 min and 1, 3, and 6 h after stimulation. (**b**) Cells migrating to the scratched area, as shown in panel A, were measured and plotted in graphs (cells/pixels) at each time point. The macrophage migrations were compared among medium (green line), WT (continuous line), and CD14 KO (dotted line) macrophages and between WT and CD14 KO macrophages stimulated with ArtinM (red line). P3C4 (blue line) was used as the positive control. The data are shown as means ± SEM of independent experiments. *p < 0.05, according to one-way analysis of variance and Bonferroni’s test.

M1-polarized macrophages display enhanced effector functions such as phagocytic and microbicidal activities^38^. We hypothesized that ArtinM stimulates the migration and phagocytic activities of macrophages as it does in neutrophils^39^. The migration of macrophages obtained from WT and CD14 KO mice was assessed by a wound healing assay. Compared with unstimulated cells, macrophages stimulated with ArtinM or P3C4 showed significantly increased migration 6 h after treatment (Fig. 3a [left panels] and b). The lack of CD14 inhibited migration in macrophages stimulated by ArtinM or P3C4 (see Fig. 3a [right panels] and b); in both cases, the cells displayed migratory capabilities that were as low as those observed in unstimulated macrophages.

**Figure 4:**
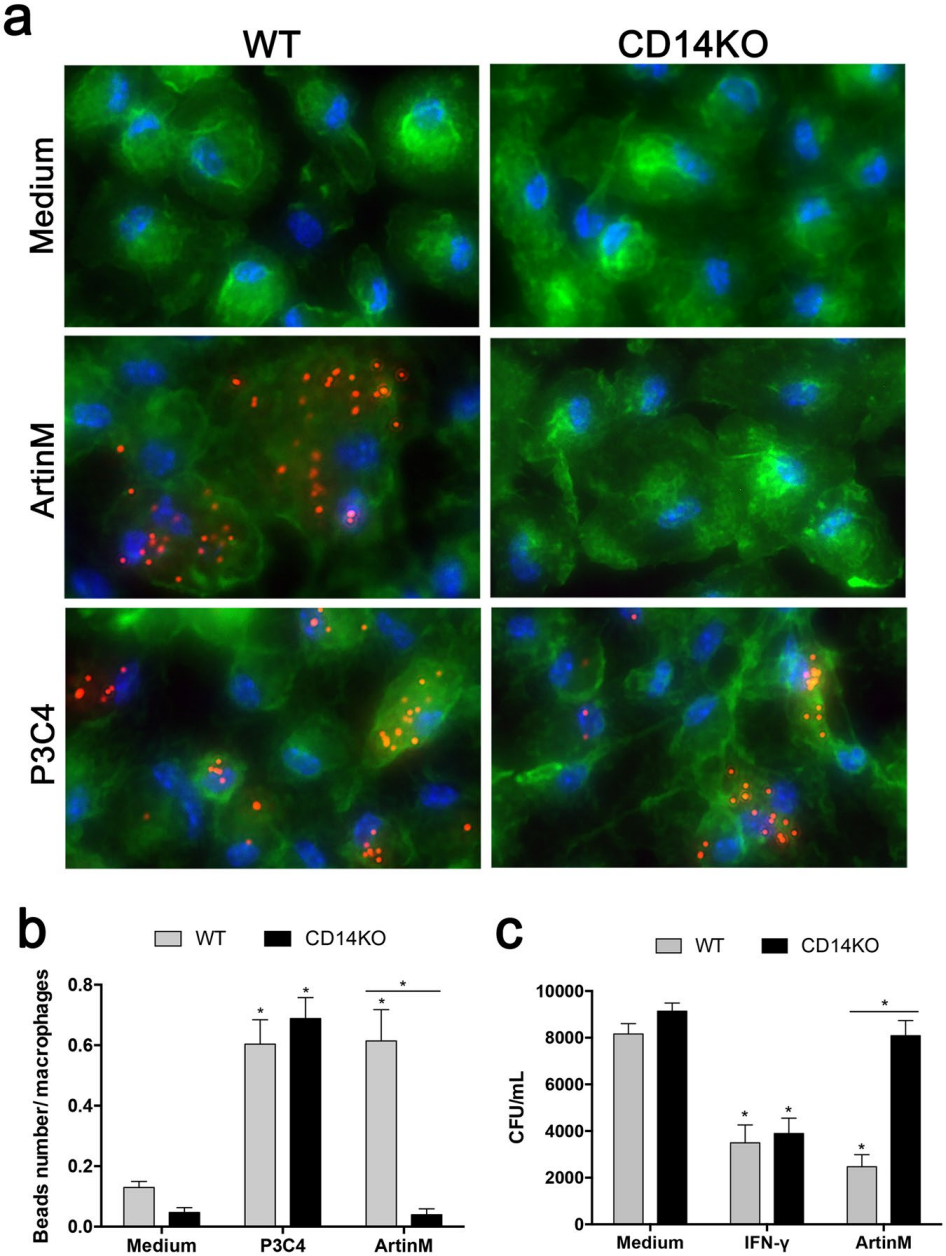
Phagocytic and fungicide activity of ArtinM-stimulated macrophages depends on CD14. (**a** and **b**) Phagocytic activity was realized in macrophages (5 × 10^5^/well) from the WT and CD14 KO mice after stimulation with ArtinM (2.5 µg/mL), P3C4 (1 µg/mL), or medium for 16 h. The cells were then incubated with phycoerythrin-conjugated latex beads (red) for 30 min, stained with phalloidin-fluorescein isothiocyanate (green) and 4ʹ,6-diamidino-2-phenylindole (blue), and processed for fluoresce microscopy (**a**). The images were acquired on a fluorescence microscope at 40× magnification and were used to measure the number of beads internalized per macrophage from the WT (gray bar) and CD14KO (black bar) mice. The mean number of beads/macrophage was plotted (**b**). (**c**) The fungicide activity assay was carried out in macrophages (1 × 10^6^/mL) from the WT and CD14KO mice after stimulation with ArtinM (2.5 µg/mL), IFN-γ (50 ng/mL), or medium. After 24 h, the macrophages were infected with P. brasiliensis (yeast-to-macrophage ratio of 1:10), for 4 h. The non-internalized yeasts were removed and the medium was added to the cells, which were cultured for an additional 48 h. The macrophages were lysed with distillated water for the measurement of viable intracellular yeasts by CFU. (**a**–**c**) The phagocytic and fungicide activity in the WT and CD14 KO macrophages were compared with those of cells in medium, and the values were also compared between the WT and CD14 KO macrophages stimulated with ArtinM, P3C4, or IFN-γ. The data are shown as means ± SEM of independent experiments. *p < 0.05, according to one-way analysis of variance and Bonferroni’s test.

The phagocytic ability was evaluated by quantitating fluorescent latex beads engulfed by ArtinM-stimulated macrophages. Using fluorescence microscopy to count intracellular beads showed that, compared with the unstimulated control cells, WT macrophages stimulated with ArtinM or P3C4 phagocytized four times more beads per cell (Fig. 4a [upper panels] and b [gray bars]). When CD14KO macrophages were tested, the phagocytic capability stimulated by P3C4 was preserved, whereas ArtinM stimulation was associated with a phagocytic capacity as small as that in unstimulated cells (see Fig. 4a [lower panels] and b [black bars]). These results indicate that ArtinM stimulates the migratory and phagocytic effector functions of macrophages through mechanisms that depend on CD14.

Because macrophages with an M1 phenotype are known to display a strong microbicidal capacity^38, 40^ we determined whether ArtinM-stimulated macrophages could promote the killing of *P. brasiliensis*. Macrophages from WT and CD14KO mice were stimulated for 24 h with IFN-γ, ArtinM, or medium. Subsequently, the macrophages were incubated with *P. brasiliensis* for 4 h, the non-internalized yeasts were removed, and the macrophages were incubated for 48 h at 37 °C. The colony-forming unit (CFU) recovery from the macrophage lysate showed that stimulation with either IFN-γ or ArtinM was associated with CFU counts lower than those obtained from unstimulated macrophages (Fig. 4c). However, after stimulation with ArtinM the CD14KO macrophages showed CFU recovery at levels closer to those of unstimulated macrophages (Fig. 4c). Thus, we concluded that ArtinM induces high fungicidal activity in macrophages in the presence of CD14.

### CD14 modulates the level of TLR2 expressed by ArtinM-stimulated macrophages

**Figure 5:**
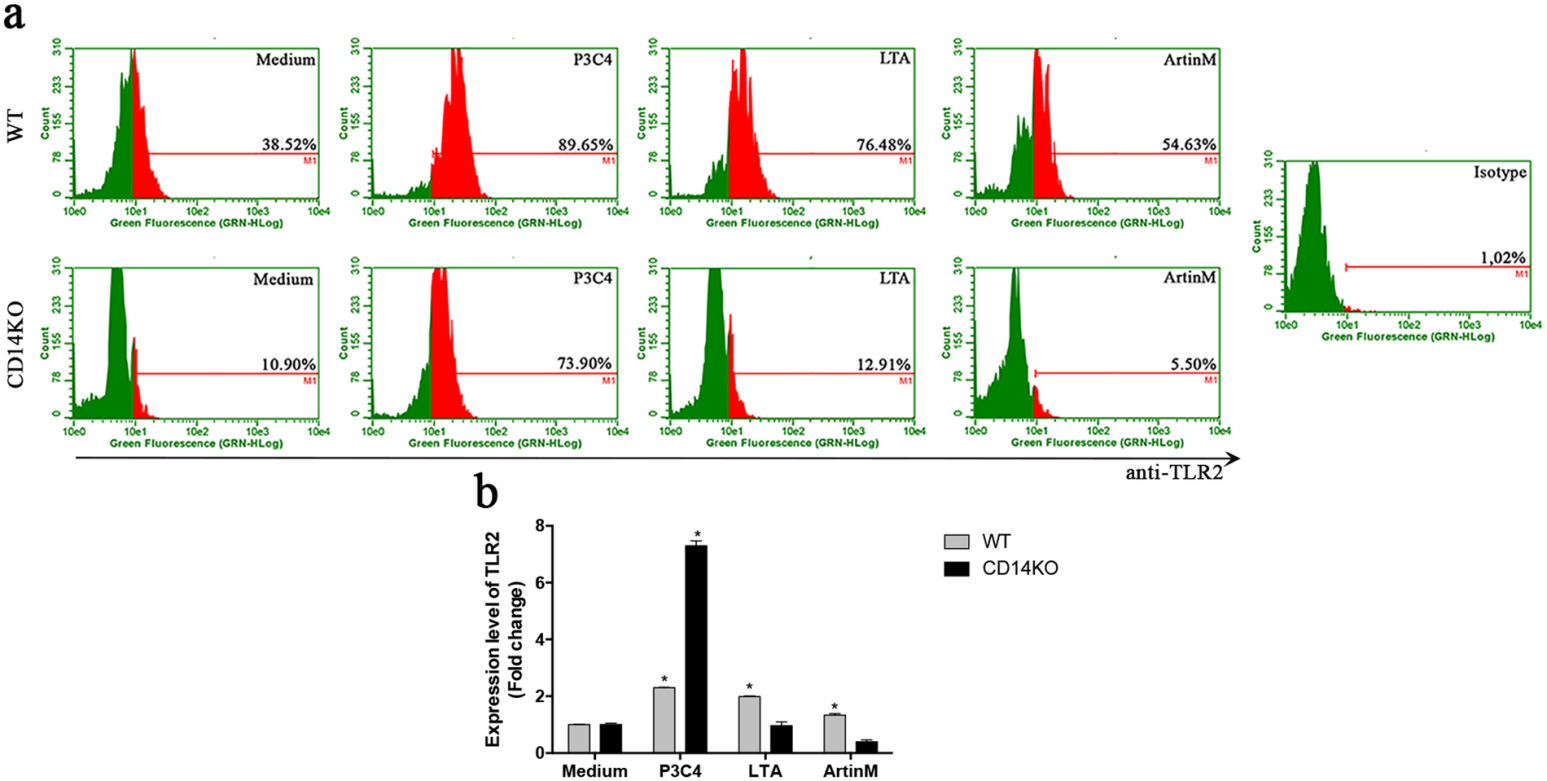
Expression of TLR2 in ArtinM-stimulated macrophages decreases in the absence of CD14. Macrophages (1 × 10^6^/mL) from the WT and CD14 KO mice were stimulated with ArtinM (2.5 µg/mL), P3C4 (1 µg/mL), LTA (1 µg/mL), or medium for 48 h. The macrophages were incubated with anti-TLR2 antibody (15 µg/mL), and the fluorescence was measured by flow cytometry. (**a**, histogram) The percentage of fluorescent cells stained for TLR2 was determined for each condition, and (**b**, bars) was calculated the fold change of the TLR2 expression under stimuli compared to the medium. The data are shown as means ± SEM of independent experiments. *p < 0.05, according to one-way analysis of variance and Bonferroni’s test.

Taking into consideration the importance of TLR2 and CD14 to the effects exerted by ArtinM on macrophages, we determined whether CD14 influences the expression of TLR2 in ArtinM-stimulated macrophages. To this end, murine peritoneal macrophages from WT and CD14 KO mice were stimulated *in vitro* with ArtinM, P3C4, or LTA, and assessed for TLR2 expression by flow cytometry. The results expressed as a percentage of positive cells showed that the ArtinM-stimulated WT macrophages exhibited a significant increase in TLR2 expression compared with cells in the medium (see Fig. 5a [histogram] and b [gray bars]). Notably, in CD14 KO macrophages stimulated with ArtinM, the expression of TLR2 was drastically downregulated to levels as low as those observed in the negative control (see Fig. 5a [histogram] and b [black bars]). TLR2 expression was similarly induced by P3C4 in WT and CD14 KO macrophages, whereas TLR2 expression triggered by LTA in CD14 KO macrophages was closer to that in the medium (Fig. 5). These results indicate that both ArtinM and the positive control LTA enhance the expression of TLR2 in a CD14-dependent manner.

Because we detected a lower TLR2 expression on CD14KO than on WT macrophages (Fig. 5a), we supposed that the lower activation of CD14KO macrophages by ArtinM could be a consequence of the lower TLR2 expression. Then, LPS-primed peritoneal macrophages from WT and CD14KO mice were analyzed by flow cytometry regarding the TLR2 expression levels; they were verified similar to the detected on macrophages that were just LPS-primed (Fig. S3a). These cells were then stimulated with ArtinM, P3C4, or medium. The cultures supernatants were used to determine the IL-12 production, and the LPS-primed WT macrophages showed a significant increase in the levels of IL-12 in the presence of ArtinM compared to the medium (Fig. S3a). However, the LPS-primed CD14KO macrophages stimulated with ArtinM did not differ in the IL-12 production in comparison to medium alone (Fig. S3b). These results reinforce the importance of the ArtinM interaction with CD14 to induce the macrophage activation.

### CD14 accounts for the activation of NF-κB in ArtinM-stimulated macrophages

**Figure 6:**
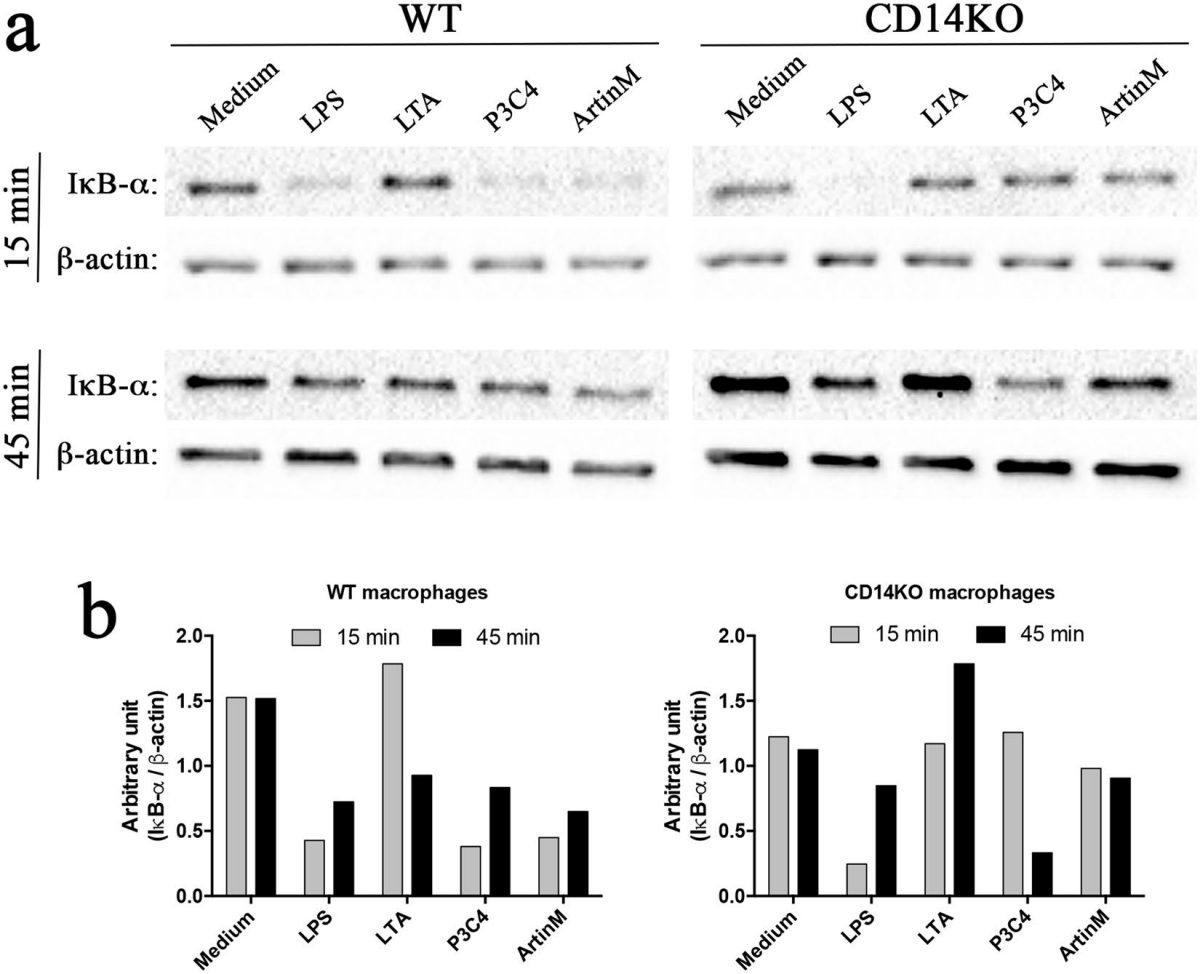
ArtinM-induced IκB-α degradation is impaired in the absence of CD14. (**a** and **b**) Macrophages (2 × 10^6^/mL) from the WT (left panel) and CD14 KO (right panel) mice were stimulated with ArtinM (2.5 µg/mL), LPS (1 µg/mL), P3C4 (1 µg/mL), LTA (1 µg/mL), or medium alone for 15 and 45 min. The cells were lysed in sample buffer and analyzed by SDS-PAGE. The electrophoresed samples were blotted onto nitrocellulose membranes and probed with anti-IκB-α antibody (clone C-21), or anti-β-actin (clone C4). (**b**) Measurement of densitometry analysis after staining of IκB-α and β-actin to normalize the IκB-α degradation compared to endogenous control. The ratio between IκB-α- and β-actin was calculated, and the values were expressed as an arbitrary unit. The data are shown as means ± SEM of independent experiments. *p < 0.05, according to one-way analysis of variance and Bonferroni’s test. The full-length WB is included in supplementary information.

We showed that ArtinM stimulated the following macrophage responses in a CD14-dependent manner: production of pro-inflammatory mediators, M1 polarization, migration, phagocytosis, fungicide activity, and TLR2 expression. We also evaluated the involvement of NF-κB, which is directly linked to TLR activation. To address this question, we examined the time-lapsed ArtinM-, LPS-, P3C4-, or LTA-induced activation of IκB-α degradation in WT and CD14 KO macrophages. These cells were used to carry out western blotting after stimulation for 15 and 45 min to analyze IκB-α degradation (Fig. 6a), and we carried out densitometry analysis to measure the ratio of IκB-α to β-actin (Fig. 6b). The WT macrophages stimulated for 15 and 45 min with ArtinM showed a significant increase in IκB-α degradation (Fig. 6a,b, left panel), and the time course of IκB-α degradation was also influenced by TLR agonists such as LPS, P3C4, and LTA. In contrast, the CD14 KO macrophages did not show IκB-α degradation 15 and 45 min after ArtinM stimulation (Fig. 6a,b, right panel), and these cells exhibited a significant decrease in the staining density of IκB-α only after LPS stimulation. To corroborate these findings, we evaluated the phosphorylation of IκB-α that results in the release and nuclear translocation of active NF-κB. We found that stimulation with ArtinM for 45 min induced phosphorylation of IκB-α in WT macrophages, but not in CD14 KO macrophages (Fig. S4). These data indicate that the interaction of ArtinM with TLR2 and CD14 promotes macrophage activation via the NF-κB pathway.

### Targeting of N-glycans determines the interaction of ArtinM, CD14, and TLR2

**Figure 7:**
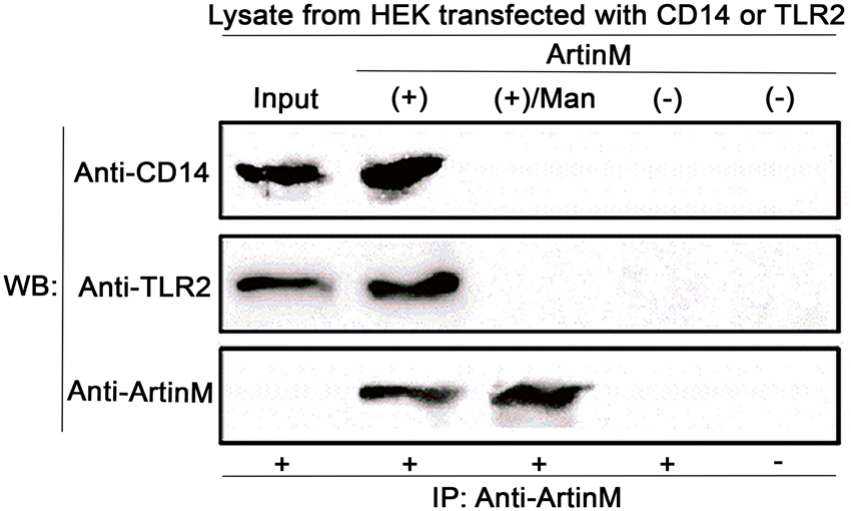
ArtinM binding with CD14 by carbohydrate recognition. Protein lysate (100 µg) from HEK cells transfected with CD14 or TLR2 was assayed. The protein lysate was incubated [+] or not [−] with ArtinM (10 µg) for 16 h at 4 °C. The protein lysate samples had been preincubated or not with Manα1-3(Manα1-6)Man [Man]. Then, 10 µg purified anti-ArtinM immunoglobulin [IP: anti-ArtinM] was added [+] or not [−] and the mixture was centrifuged and washed with ice-cold acetone. Precipitated material was analyzed with WB by anti-CD14, anti-TLR2, or anti-ArtinM antibodies.

The effects exerted by ArtinM on macrophages elucidated the role CD14 plays in the cell activation induced by the lectin. To evaluate the possible interaction with CD14 and TLR2, we performed a lectin immunoprecipitation assay in which ArtinM the lysates of human embryonic kidney (HEK) cells that were transfected with CD14 or TLR2 were incubated with ArtinM. Precipitation was performed by adding anti-ArtinM antibody, and the western blot analysis of the precipitated material revealed positive staining for CD14 and TLR2. Notably, CD14 and TLR2 were not detected when the procedure was carried out with ArtinM that was pre-incubated with its trimmanoside ligand. ArtinM interaction with CD14 and TLR2 was considered specific because these proteins were not detected when ArtinM or anti-ArtinM was omitted from the procedure (-ArtinM or -Anti-ArtinM, respectively; Fig. 7).

## Discussion

Macrophage activation induced by ArtinM was originally related to the interaction of the lectin with TLR2, which was found to occur in a carbohydrate recognition-dependent manner^31^; this recognition is responsible for the immunomodulatory activity of ArtinM that accounts for the protection conferred on *P. brasiliensis*-infected mice^27, 28^. Here, we demonstrated that ArtinM also interacts with CD14, thereby establishing a process that is critical for the effects of the lectin on macrophages, such as the production of proinflammatory mediators, M1 polarization, migration, and enhancement of phagocytic and fungicide activity. We also showed that ArtinM binding to CD14 initiates macrophage activation via the NF-κB pathway, which leads to the upregulation of TLR2. Therefore, we postulate that CD14 N-glycans are primary docking sites for ArtinM, which further interacts with TLR2 N-glycans and activates macrophages toward an inflammatory profile.

We based the choice of peritoneal macrophages to perform the current study on evidences of their phenotypic and functional stability, which favors the investigation on the M1/M2 polarization induced by different agents^41^. The use of BMDMs implies in some important disadvantages, such as their unstable phenotype, associated with high expression of CD115 and Gr1, which indicates cellular immaturity^41, 42^. In addition, diminished detection of MHC II and CD86 on BMDMs is related to cells’ maturity^41, 43, 44^. Moreover, M-CSF, necessary to promote *in vitro* BMDM differentiation, favors the cell polarization into an M2 phenotype^45, 46^.

We have demonstrated that macrophage activation induced by ArtinM involves the binding of ArtinM to TLR2, but not to TLR4, which is considered a receptor that primarily engages CD14^27, 31^. Although the CD14 molecule, recognized by ArtinM, has no intracellular domain, it was previously reported that the binding of lipopeptides to TLR2 is facilitated by CD14 and promotes the assembly of a ternary complex with the relocation of TLR2 and CD14 to membrane microdomains^47^. Therefore, we hypothesized that, as a tetrameric protein, ArtinM bridges glycans that are N-linked to CD14 and TLR2 based on the synergistic physical interactions between ArtinM, TLR2, and CD14 detected herein. Moreover, the proposed ArtinM binding to CD14 is essential for macrophage activation. These activities suggest a particular functioning of CD14, the oligosaccharides of which would be targeted by a lectin, thereby favoring the assembly of the TLR2 signaling complex and, consequently, promoting cell activation. In addition, we have been evaluating the importance of CD36 molecule in the ArtinM interaction with TLR2, and our data suggest that CD36 is not essential for macrophage activation by ArtinM.

A lack of CD14 hampers the macrophage response to LPS^48^ and the TLR2 agonist lipoteichoic acid^49, 50^. Consistent with the reports that TLR2-mediated responses can be enhanced by CD14^47, 51^, we found that in its absence macrophage activation was partially affected in response to P3C4, and was abolished following stimulation by LTA. A TLR2/CD14-dependent response has been reported in macrophages stimulated with LTA from *Staphylococcus aureus* ^50^ and *Enterococcus faecalis* ^49^. Recently, a virulence factor of *Bacillus anthracis*, namely poly-γ-D-glutamic acid (PGA), was found to stimulate the expression of proinflammatory cytokines through a pathway that involves TLR2/TLR6/CD14 activation^52^. The dependence on CD14 may vary according the assayed agonist and its concentration, as reported for distinct bacterial LPS molecules^53^. Considering the effects of ArtinM on cytokine production (IL-12p40, TNF-α, IL-6, and IL-10) by macrophages, the presence of CD14 is indubitably essential. Among the three plant lectins other than ArtinM shown to induce IL-12 production by macrophages (ConA, L-PHA, and E-PHA), only ConA activity was slightly affected by the absence of CD14. In addition, we identified in a different source of macrophages, namely CD11b^+^ cells purified from spleen cells, that the IL-12 production induced by ArtinM in WT CD11b^+^ cells was abolished in the absence of CD14 (Fig. S3c). A conspicuous dependence on CD14 was displayed only by ArtinM, which indicates its affinity toward particular glycan structures of this co-receptor. Although ArtinM recognizes primarily Manα1-3(Manα1-6)Man, which is the common core of N-glycans, the fine specificity of its binding is further determined by a secondary subsite of recognition. It interacts with other carbohydrate residues (Fuc and GlucNAc), which may be contained in the N-glycan, resulting in the particular binding property exhibited by this lectin^32, 33, 54^.

We demonstrated here that, in the presence of CD14, ArtinM induces an inflammatory profile in macrophages with robust iNOS2 expression, NO production, and IL-12 production, accompanied by IL-10 release. Previous studies have verified that LTA from *S. aureus* ^50^, *Staphylococcus mutans* ^55^, *E. faecalis* ^49^, *Lactobacillus fermentum* ^56^, and *Lactobacillus casei* ^56^ induces the production of NO and/or proinflammatory cytokines, in a manner that depends on concentration, because the lowest concentrations (0.1–1.0 μg/mL) did not effectively promote the release of proinflammatory mediators. Consistently, we found that LTA from *S. aureus*, used at a concentration of 1.0 μg/mL in our experiments, did not induce M1 polarization of the WT macrophages. Despite this, LTA-stimulated WT macrophages enhanced the production of proinflammatory cytokines. We found that stimulation with P3C4 induced the M1 polarization of WT macrophages, whereas a lack of CD14 did not significantly affect the production of proinflammatory cytokines. Concerning the response to ArtinM, we found that the CD14-deficient macrophages did not undergo the same polarization and cytokine production as the WT macrophages. Moreover, ArtinM induced IL-10 production by WT macrophages, a response that was not detected in CD14 KO macrophages. It is known that classic M1 macrophages are expected to produce low levels of IL-10 because this cytokine inhibits IL-12 transcription and promotes the expression of c-Maf, a selective transcriptional inhibitor of IL-12p40 and p35 genes^57^. The mechanism underlying the development of an M1 profile with joint IL-10 production remains unknown.

NF-κB activation triggered by TLR engagement leads to the production of inflammatory mediators associated with phagocytic cell migration^58^ and M1 polarization of macrophages^59^. The inhibition of NF-κB activation also blocks the M1 phenotype and tumoricidal activity of bone marrow-derived macrophages^60^. Moreover, the production of nitric oxide by iNOS is associated with the induction of Src expression and activity, which leads to focal adhesion kinase activation and macrophage mobility^58^. An additional mechanism has been proposed for LPS-mediated macrophage migration, which occurs in the absence of chemoattractants; it involves increased phosphorylation of two actin-regulatory proteins—paxillin and neural Wiskott–Aldrich syndrome protein—before NF-κB activation^61^. Notably, CD14 enhances the phagocytosis of apoptotic cells^62^ by interacting with CR3 (β2-integrin). In spirochetal infection, increased phagocytosis occurs in a manner that is independent of TLR/MyD88-induced signals^63^. We have verified that ArtinM stimulates the migratory and phagocytic activities of neutrophils^39, 64^, as well as their microbicidal ability^65^. These effects have not been reported for macrophages, and we showed herein that ArtinM promotes the migration of macrophages and their phagocytic and fungicidal activities, a response that did not occur in cells from CD14 KO mice.

The induction of cytokine production, cell motility, and phagocytosis involves the NF-κB pathway, which is triggered by TLR^66^. NF-κB is located in the cytoplasm and interacts with inhibitor factors, known as IκB proteins. They are rapidly phosphorylated following TLR activation, becoming targets for ubiquitination and subsequent degradation^66, 67^. The authors of previous studies have reported that ArtinM promotes the transcription of NF-κB target genes in mast cells^68^. Therefore, we investigated the association between the ArtinM-induced activation of macrophages and the NF-κB pathway through measurement of IκB degradation. We showed herein that macrophage activation triggered by ArtinM is associated with that signaling pathway and depends on the lectin interaction with CD14. Several microbial products are recognized by either membrane-bound or soluble CD14^69^, and promote TLR2 or TLR4 engagement, which results in the activation of the NF-κB pathway^66^. We verified the induction of the NF-κB pathway in CD14 KO macrophages when stimulated with LPS, a TLR4 agonist that activates several intracellular kinases in a CD14-dependent manner^70^. Therefore, it is plausible that similar activation of macrophages may result from lectin binding to cell surface receptors. Consistently, lectins such as ConA, wheat germ agglutinin (WGA), and PHA activate the NF-κB pathway in T cells^71^.

The subsequently triggered intracellular cascades led to the upregulation of TLR2 expression, which was increased after stimulation with ArtinM in a CD14-dependent manner. In this way, neutrophils upregulate TLR2 expression after stimulation by ArtinM^39^, and the expression of TLR2 could be controlled by inflammatory regulators^72^ as well as by some infections^73^. Accordingly, higher TLR2 expression may create an activation loop that improves cellular response to new lectin molecules and secreted inflammatory mediators. In addition, our data on the manifestations of ArtinM-induced cell activation, in which CD14 cooperates, indicate that the studied process of cell activation is a multi-receptor phenomenon and provides novel knowledge about the biological repercussions of lectin interactions. In this sense, we demonstrated that TLR2 agonists, like P3C4 and LTA, modulate the levels of TLR2 expression, which could be dependent on CD14.

Currently, new drugs are being developed based on TLR agonists to improve immunity. This kind of host-directed immunotherapy is promising for combating infectious diseases because it accommodates pathogen evolution instead of causing microbial resistance, as occurs with antibiotics^23, 74^. Also, the importance of carbohydrate recognition in biology motivates a growing interest in mimicking lectins. Although only a few synthetic lectins operate in water and are truly biomimetic, supramolecular chemists do not categorize the mimicry of lectins as unrealistic^37^. Therefore, ArtinM interaction with TLR2 and CD14 acquires particular relevance when we consider the capability of a few lectins to efficiently modulate adaptive immunity toward the Th1 axis. This modulation confers protection against several intracellular pathogens. Given the biological relevance of the ArtinM interactions observed in our studies, we believe that synthetic ArtinM has potential as an agent for modulating immunity and preventing or treating infectious disease.

## Materials and Methods

### Mice

C57BL/6 (WT) and CD14 KO (C57BL/6 genetic background) mice (6–8 weeks old), acquired from the animal facility of the Ribeirão Preto Medical School at the University of São Paulo, were bred and housed under optimized hygienic conditions at the animal facility of the Molecular and Cellular Biology Department of the Ribeirão Preto Medical School. All protocols were approved by the Committee on Ethics in Animal Research of the Ribeirão Preto Medical School at the University of São Paulo and in accordance with the Ethical Principles in Animal Research adopted by the Brazilian College of Animal Experimentation, Protocol 088/2010.

### ArtinM

ArtinM was purified as described previously^24^ from the saline extract of *Artocarpus heterophyllus* (jackfruit) seeds through affinity chromatography on immobilized carbohydrate columns. Before use, ArtinM aliquots were incubated for 1 h with a polymyxin solution (50 μg/mL; Sigma-Aldrich, St. Louis, MO, USA).

### Production of pro-inflammatory mediators by macrophages

The levels of IL12p40, TNF-α, IL-6, and IL-10 produced by thioglycollate-elicited macrophages harvested from C57BL/6 (WT) or CD14 KO mice were determined 48 h after incubation at 37 °C in a humidified atmosphere containing 5% CO_2_. ArtinM (2.5 µg/mL), ConA (10 µg/mL), L-PHA (10 µg/mL), E-PHA (10 µg/mL), MAL (10 µg/mL), SNA (10 µg/mL), Jacalin (10 µg/mL), P3C4 (1 µg/mL), LTA (1 µg/mL), or medium were added to the macrophages. The levels of IL-12p40 were also measured in CD11b^+^ cells purified (Miltenyi Biotec) from spleen cells from WT and CD14KO mice, after the incubation with ArtinM (2.5 µg/mL), P3C4 (1 µg/mL), or medium alone for 48 h. The levels of cytokines were measured in the culture supernatant using enzyme-linked immunosorbent assay (ELISA) kits (BD Biosciences, San Diego, CA, USA) according to the manufacturer’s protocol.

Nitric oxide concentration was inferred by measuring nitrite levels in the macrophage supernatants using the Griess reagent system^75^. The absorbance at 540 nm was read using a Power Wave-X microplate reader (BioTek Instruments, Inc.). The absorbance was converted to micromolar (µM) NO on the basis of a standard curve, concomitantly generated by using known concentrations of NaNO_2_.

### Quantitative reverse transcription PCR

RNA from macrophages stimulated for 10 h was isolated by using the TRIzol Reagent (Life Technologies, Carlsbad, CA, USA) according to the manufacturer’s protocol. The total RNA was reverse-transcribed into complementary DNA (cDNA) by using the ImProm-II Reverse Transcription System (Promega, Fitchburg, WI, USA) and oligo (dT) primers. Real-time PCR was performed in 15 µL reactions by using SYBR Green (Applied Biosystems/Life Technologies, Carlsbad, CA, USA), cDNA (40–100 ng), and 0.3 µM of the primer mix. All reactions were performed on a 7500 Real-Time PCR System (Applied Biosystems) with the following conditions: 50 °C for 2 min, 95 °C for 2 min, and 40 cycles of 95 °C for 15 s/60 °C for 1 min. Gene expression was quantified by using the ΔΔCt method and normalized to β-actin expression. The PCR primers used were: β-actin (F-CCTAAGGCCAACCGTGAAAA, R-GAGGCATACAGGGACAGCACA), Ym1 (F-TCACAGGTCTGGCAATTCTTCTG, R-ACTCCCTTCTATTGGCCTGTCC), Arginase-1 (F-GTTCCCAGATGTACCAGGATTC, R-CGATGTCTTTGGCAGATATGC), FIZZ1 (F-CCTGAGATTCTGCCCCAGGAT, R-TTCACTGGGACCATCAGCTGG), and iNOS2 (F-CCGAAGCAAACATCACATTCA, R-GGTCTAAAGGCTCCGGGCT).

### Wound healing assay

An *in vitro* scratch-wound healing assay was used to study cell migration. Macrophages were seeded in 24-well plates (1 × 10^6^/well), and after adhesion, linear scratch wounds were made in the cell monolayers with a 200-µL pipette tip. The cells were then stimulated with ArtinM, and the scratch wounds were visualized with a phase contrast light microscope (Nikon TS100). Photomicrographs were captured using a Leica DC300F camera (Leica, Wetzlar, Germany) 10 min and 1, 3, and 6 h after stimulation. The area of each scratch wound was determined by using ImageJ software.

### Phagocytosis and fungicide assay

Macrophages (5 × 10^5^/well) were applied to 13-mm round coverslips placed on 24-well plates. After 16 h of stimulation with ArtinM (2.5 μg/mL), P3C4 (1 μg/mL), or medium, the fluorescent beads were added to the cells (Sigma-Aldrich; 15 beads/cell) and incubated for 30 min. Subsequently, the cells were washed with phosphate-buffered saline (PBS), fixed with 2% paraformaldehyde, and permeabilized with 0.3% Triton X-100. Finally, the coverslips were stained with phalloidin-Alexa 488 (Life Technologies) and mounted on a microscope slide with Prolong Gold antifade reagent 4′,6-diamidino-2-phenylindole (Invitrogen Life Technologies, Carlsbad, CA, USA). The images were acquired on a fluorescence microscope Eclipse e800 (Nikon Instruments, Tokyo, Japan) using 20× and 40× magnification with a Nikon DXM 1200 digital camera (Nikon). Quantification of the intracellular beads by fluorescence microscopy was performed on five images acquired in each group (approximately 100 cells/image).

The fungicidal activity of macrophages was examined by colony forming units (CFU) recovery of *P. brasiliensis*. The macrophages (1 × 10^6^/mL; 48-well plates) were previously treated with ArtinM (2.5 μg/mL), IFN-γ (50 ng/mL), or medium for 24 h. Subsequently, the culture supernatant was removed and the cells were incubated for 4 h with *P. brasiliensis* (yeast to macrophages ratio of 1:10). After washing, the medium was added to the macrophages, and the cells were maintained in culture for 48 h. The measurement of CFU was carried out after macrophage lysis with distilled water (100 μL); the suspension was plated in brain heart infusion (BHI) medium to quantify the presence of viable yeasts by CFU.

### TLR2 expression by flow cytometry

Macrophages (2 × 10^6^/mL) from WT and CD14KO mice were distributed in a 48-well microplate and cultured in the presence of ArtinM (2.5 µg/mL), P3C4 (1 µg/mL), LTA (1 µg/mL), or medium. The cells were incubated for 48 h and the wells were rinsed with ice-cold phosphate-buffered saline (PBS) to detach the macrophages. After washing with PBS, the cells were incubated for 20 min at 4 °C with 0.5 µg of Fc block (clone 2.4G2, BD Pharmingen, San Diego, CA). After washing, anti-TLR2-FITC antibody (10 µg/mL; clone 6C2, eBioscience) was added and incubated for 45 min at 4 °C. The cells were washed twice with PBS and fixed with 1% formaldehyde-PBS. We used flow cytometry (Guava easyCyte) to evaluate the fluorescent cells, and determined the percentage of positive cells for each experimental condition.

### Western blotting

We stimulated macrophages (2 × 10^6^/mL) from the WT and CD14 KO mice with ArtinM (2.5 µg/mL), LPS (1 µg/mL), P3C4 (1 µg/mL), LTA (1 µg/mL), or medium for 15 and 45 min. Subsequently, the cells were washed in cold PBS and lysed with RIPA buffer (150 mM sodium chloride, 1.0% Triton X-100, 0.5% sodium deoxycholate, 0.1% sodium dodecyl sulfate (SDS), 50 mM Tris, pH 8.0) supplemented with protease and phosphatase inhibitors. Lysate samples were analyzed by SDS-polyacrylamide gel electrophoresis and transferred onto nitrocellulose membranes following a protocol described elsewhere^76^. The membrane was probed with anti-IκB-α (1:1000; clone C-21, Santa Cruz, CA, USA), anti-p- IκB-α (1:1000; clone B-9, Santa Cruz, CA, USA) and anti-β-actin (1:1000; clone C4, Santa Cruz, CA, USA). We then carried out a second staining with peroxidase-conjugated secondary antibody (1:3000). The membrane was incubated with ECL reagent for 1 min to detection of protein using a ChemiDoc MP Imaging System (Bio-Rad, Hercules, USA).

### Vectors and TLR2 FLAG cloning

pCDNA3.1 CD14 and pCDNA4 FLAG/TO vectors were kindly provided by Dr. Igor C Almeida^77^ and Dr. Dario Simões Zamboni, respectively. The pCDNA4 FLAG TLR2 vector was constructed as follows. RNA from a P388 cell line was extracted and converted to cDNA with Maxima H Minus Reverse Transcriptase (Thermo-Fisher Scientific, Waltham, MA USA) and oligo(dT). TLR2 cDNA was obtained via amplification from total cDNA by using Phusion High-Fidelity DNA Polymerase and the phosphorylated primers TLR2 F: ATGCTACGAGCTCTTTGGCTCTTCTGG and TLR2 R: CTAGGACTTTATTGCAGTTCTCAGATTTACCCAAAAC. The 2355-bp fragment was isolated from a 0.8% agarose/Tris-acetate-ethylenediaminetetraacetic acid gel, purified with a GeneJET Gel Extraction Kit (Thermo-Fisher Scientific), and inserted into the pCDNA4 FLAG/TO vector previously linearized via inverse PCR by using the primers pCDNA4 FLAG F: GGATCCACTAGTCCAGTGTGGTGGAA and pCDNA4 FLAG R: CTTGTCATCGTCATCCTTGTAATCGATGTC. The vector was treated with *Dpn*I enzyme (Thermo-Fisher Scientific). Ligation reaction was performed by using a 10:1 insert/vector ratio and T4 DNA Ligase (Thermo-Fisher Scientific) and transformed into chemically competent *Escherichia coli* DH5α cells. Proper transformants were isolated from LB agar medium plates under ampicillin selection and analyzed with PCR, restriction fragment analysis, and DNA sequencing. All reactions were performed according to the manufacturer’s instructions.

### HEK (PEAK^stable^) cell transfection and protein lysate preparation

HEK (PEAK^stable^) cells from an 80% confluent culture were seeded in 100-mm plate dishes (3 × 10^6^ cells/plate) and cultured in RPMI 1640 medium supplemented with 10% fetal bovine serum at 37 °C in a humidified atmosphere with 5% CO_2_ for 24 h. Cells were transfected with 12 µg CD14- or TLR2-coding plasmids by using polyethylenimine (1 mg/mL) in a 1:3 DNA/polyethylenimine ratio. Cells were incubated with liposomal complex for 6 h as described above. After transfection, the culture media was replaced, and the cells were cultured for 48 h under the same conditions.

To obtain the protein lysate, we detached the cells from the plates with ice-cold PBS followed by two washings for serum removal (300 × *g*, 4 °C for 10 min). Pelleted cells were lysed via addition of non-denaturing lysis buffer (20 mM Tris, pH 8.0, 137 mM NaCl, 2 mM ethylenediaminetetraacetic acid) supplemented with protease inhibitor. After 30 min of incubation on ice, the protein lysate was clarified via centrifugation (16,000 × *g*, 4 °C for 15 min). Its protein content was measured by using the bicinchoninic acid (BCA) method. The preparation was aliquoted and stored at −80 °C.

### Immunoblot analysis of ArtinM-precipitated lysates of HEK293 cells transfected with CD14 or TLR2

Lectin precipitation was performed as described by Juliano *et al*.^78^. Briefly, lysate samples containing 100 µg protein and obtained from HEK cells transfected with CD14 or TLR2, were incubated for 16 h at 4 °C with 10 µg ArtinM, either pre-incubated or not with Manα1-3(Manα1-6)Man (1 mM). Subsequently, 10 µg purified anti-ArtinM immunoglobulin Y was added and incubation was performed under similar conditions. The mixture was centrifuged at 16,000 × *g* at 4 °C, then washed twice with two volumes of ice-cold acetone. The precipitated material was maintained at room temperature for acetone evaporation, dissolved in SDS-polyacrylamide gel electrophoresis sample buffer, heated for 5 min at 100 °C, and assayed by western blotting analysis. Anti-TLR2 (clone S-16) and anti-CD14 (clone F09) were purchased from Santa Cruz Biotechnology (Santa Cruz, CA, USA).

### Statistical analysis

The results are presented as means ± standard error of the mean (SEM) for at least three experiments. The data were analyzed with Prism (Graph Pad Software) and one-way analysis of variance followed by Bonferroni’s multiple-comparison test for experiments with three treatment conditions. Differences with a p < 0.05 were considered statistically significant.

## Electronic supplementary material

Supplementary Information
