# Supplementary material for: CD14 is critical for TLR2-mediated M1 macrophage activation triggered by N-glycan recognition
Source: Sci Rep. 2017 Aug 1;7:7083. doi: 10.1038/s41598-017-07397-0 (PMC5539197; doi:10.1038/s41598-017-07397-0)

**CD14 is critical for TLR2-mediated M1 macrophage activation triggered by N-glycan recognition**

Thiago Aparecido da Silva^1*^, André L. V. Zorzetto-Fernandes^1*^, Nerry T. Cecílio^1^, Aline Sardinha-Silva^1^, Fabrício Freitas Fernandes^1^, Maria Cristina Roque-Barreira^1#^

Departamento de Biologia Celular e Molecular e Bioagentes Patogênicos, Faculdade de Medicina de Ribeirão Preto, USP, São Paulo, Brasil

# Corresponding author: Maria Cristina Roque Barreira, Departamento de Biologia Celular e Molecular e Bioagentes Patogênicos, Faculdade de Medicina de Ribeirão Preto, Universidade de São Paulo, Avenida Bandeirantes 3900, 14049-900, Ribeirão Preto, São Paulo, Brasil. Phone: +55 16 3315-3062. Fax: +55 16 3315-0728; E-mail: [mcrbarre@fmrp.usp.br](mailto:o:mcrbarre@fmrp.usp.br)

***** T.A.S and A.L.V.Z.F contributed equally to the work

### SUPPLEMENTARY INFORMATION

###
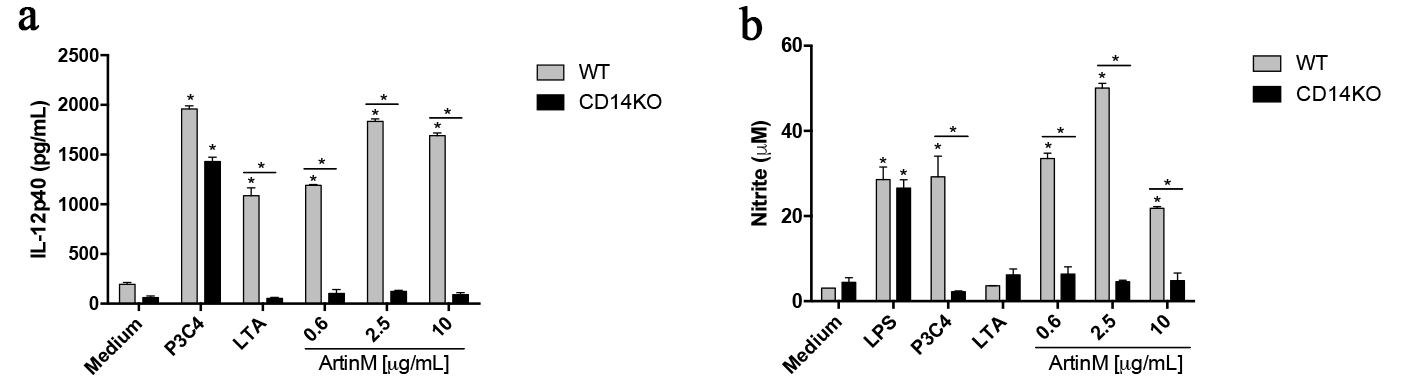


### Figure S1. Production of IL-12 and NO by CD14 KO macrophages under stimulation with different concentrations of ArtinM. Macrophages (2 × 10^6^/mL) from WT and CD14 KO mice were stimulated for 48 h with various concentrations of ArtinM (0.625 - 10 μg/mL), LPS (1 μg/mL), P3C4 (1 μg/mL), LTA (1 μg/mL) or medium alone. Culture supernatants were assessed for IL-12p40 (a), and nitric oxide (b) levels. We compared the values (in pg/mL or μM) identified in WT and CD14 KO macrophages under certain stimuli. The values were also compared to those verified in the absence of a stimulus (Medium). Data are presented as means ± SEM of independent experiments. *p < 0.05; one-way analysis of variance followed by Bonferroni’s multiple comparison test.

###
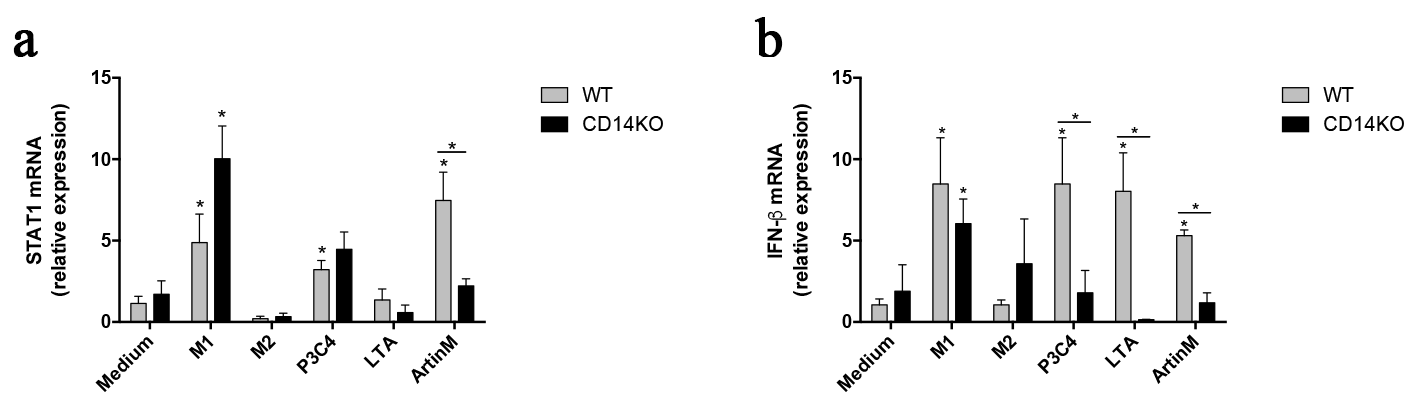


### Figure S2. ArtinM effects on STAT1 and IFN-β relative expression by macrophages depends on CD14. Macrophages (2 × 10^6^/mL) from WT and CD14 KO mice were incubated for 10 h with ArtinM (2.5 μg/mL), P3C4 (1 μg/mL), LTA (1 μg/mL), IFN-γ (M1; 50 ng/mL), IL-4 plus IL-10 (M2; 50 ng/mL), or medium only. The relative expression levels of STAT1 (a) and IFN-β (b) were determined by real- time polymerase chain reaction (PCR) in WT and CD14 KO macrophages, and the results provided by the stimulated cells were compared with those of unstimulated cells (medium only). A comparison was also established between WT and CD14 KO macrophages subjected to the effects of a particular stimulus. Data are shown as means ± SEM of independent experiments. *p < 0.05, according to one-way analysis of variance and Bonferroni’s multiple comparison test.

###
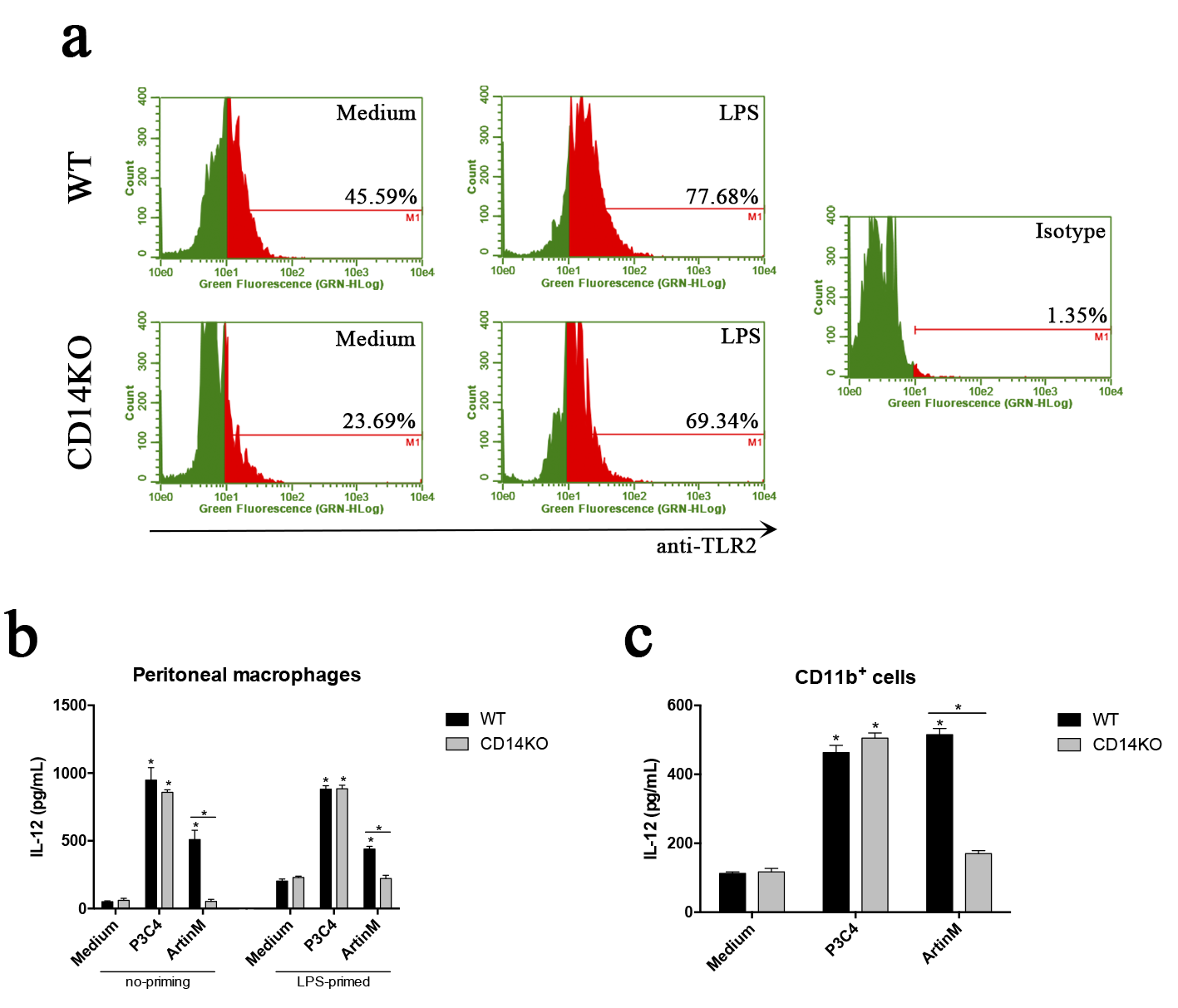


### Figure S3. LPS-primed peritoneal macrophages from WT and CD14KO mice under stimulation with ArtinM. Peritoneal macrophages (1 × 10^6^/mL) from the WT and CD14 KO mice were stimulated with LPS (LPS-primed; 0.5 µg/mL), or medium (non-primed) for 24 h. The macrophages were incubated with anti-TLR2 antibody (15 µg/mL), and the fluorescence was measured by flow cytometry (a, histogram). The percentage of fluorescent cells stained for TLR2 was determined for each condition. (b) Peritoneal macrophages, LPS-primed or non-primed, were incubated with ArtinM (2.5 μg/mL), P3C4 (1 μg/mL), or medium alone for 24 h; the IL-12p40 concentrations detected in the culture supernatants were assessed by ELISA, and the values identified in WT and CD14 KO macrophages under certain stimuli were compared. The values were also compared to those verified in the absence of a stimulus (Medium). (c) CD11b^+^ cells purified from spleen cells from WT and CD14 KO mice were stimulated with ArtinM (2.5 μg/mL), P3C4 (1 μg/mL), or medium alone, for 48 h. Data are shown as means ± SEM, and *p < 0.05 according to one-way analysis of variance and Bonferroni’s multiple comparison test.

###
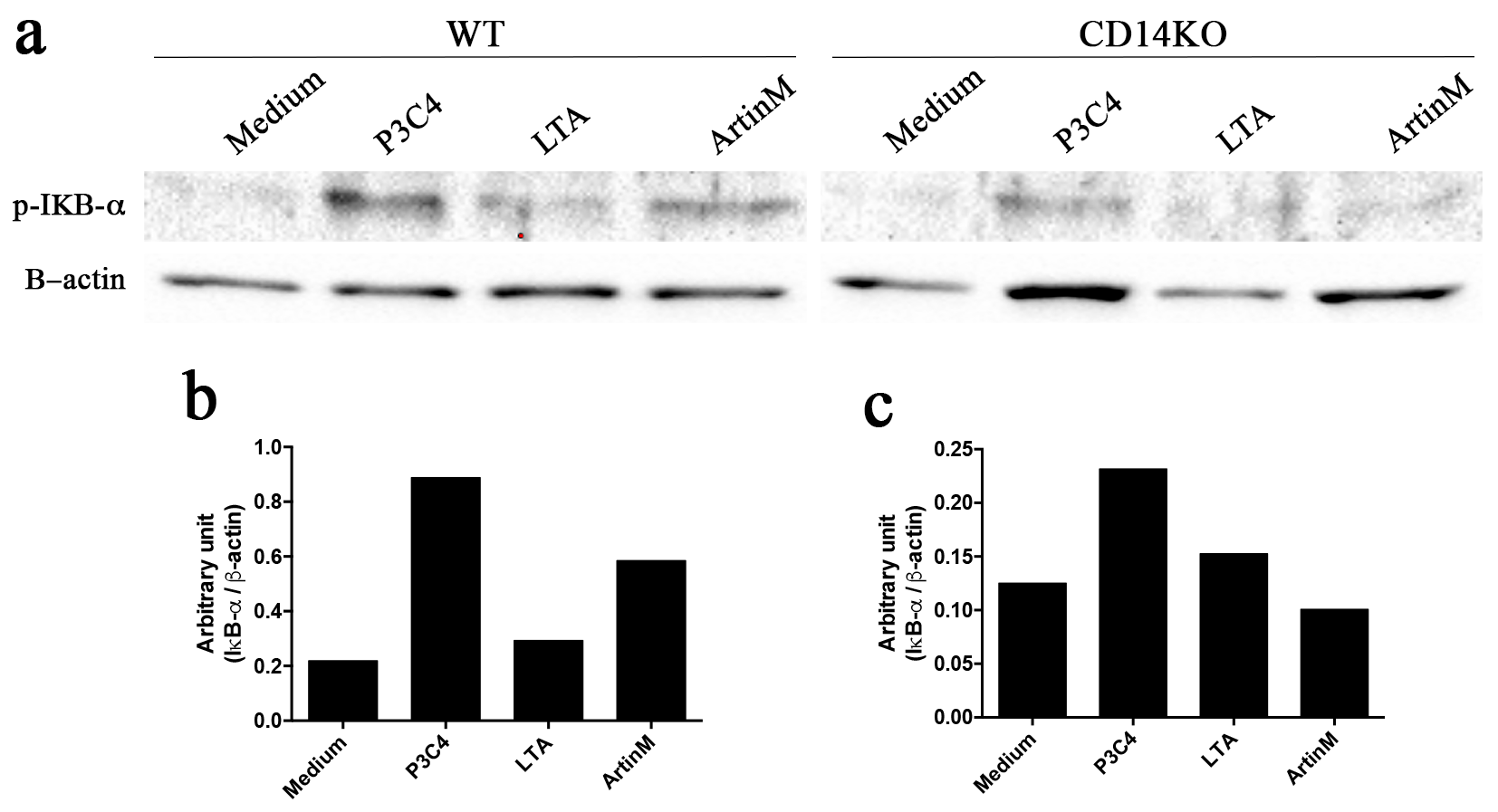


### Figure S4. ArtinM-induced phosphorylation of IκB-α in macrophages depends on lectin interaction with CD14. (a) Macrophages (2 × 10^6^/mL) from WT (left panel) and CD14 KO (right panel) mice were stimulated with ArtinM (2.5 μg/mL), P3C4 (1 μg/mL), LTA (1 μg/mL), or medium alone for 45 min. Cells lysates were analyzed by WB, which was probed with a phospho-IκB-α antibody, or anti-β-actin antibody. (b and c). Data obtained by the WB densitometric analysis, after staining of p-IκB-α and β-actin to normalize the induction of p-IκB-α compared to endogenous control. The ratio between p-IκB-α and β-actin was calculated, and the values were expressed as an arbitrary unit. The full-length WB is demonstrated in supplementary information.

###
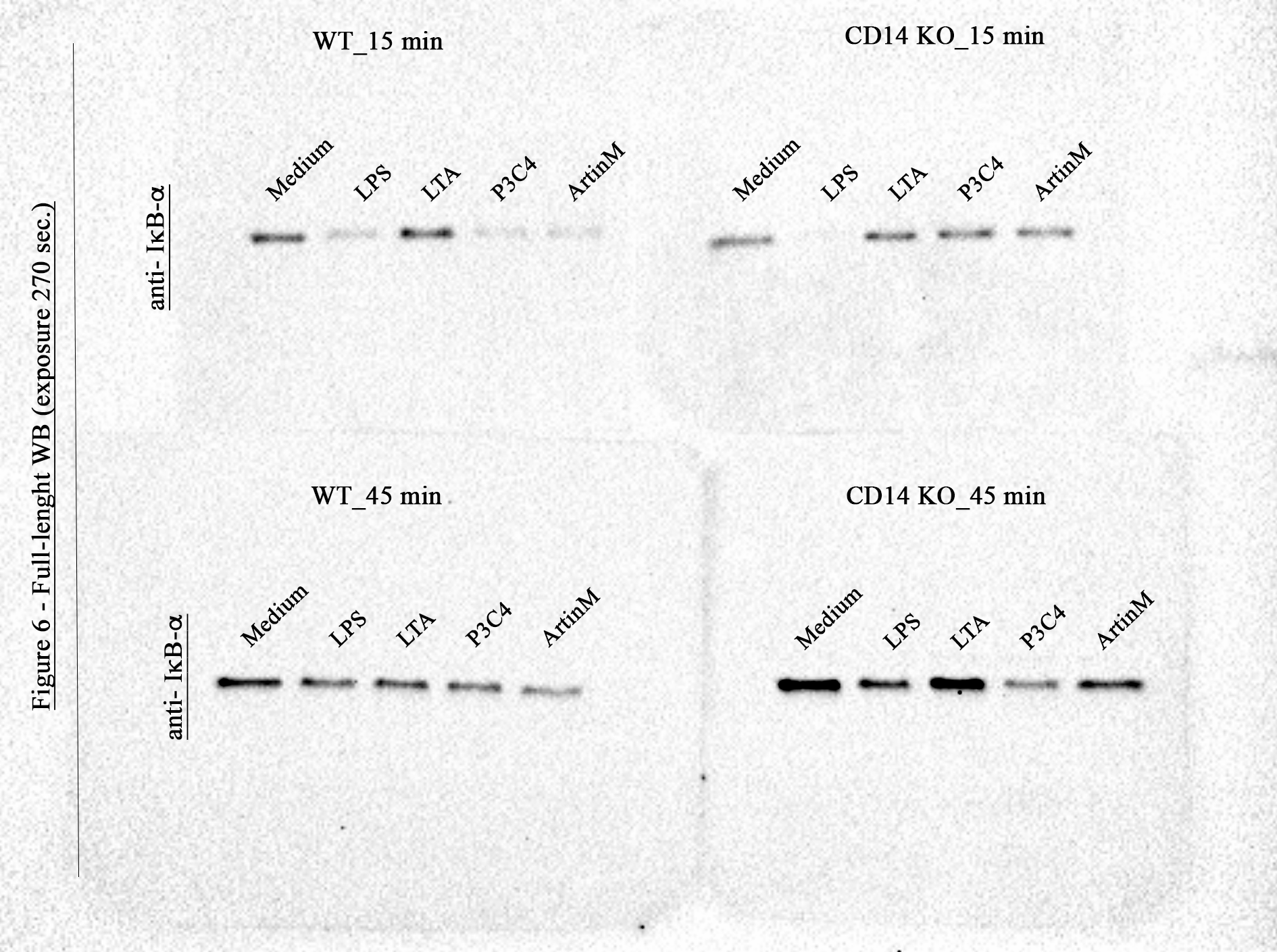


###
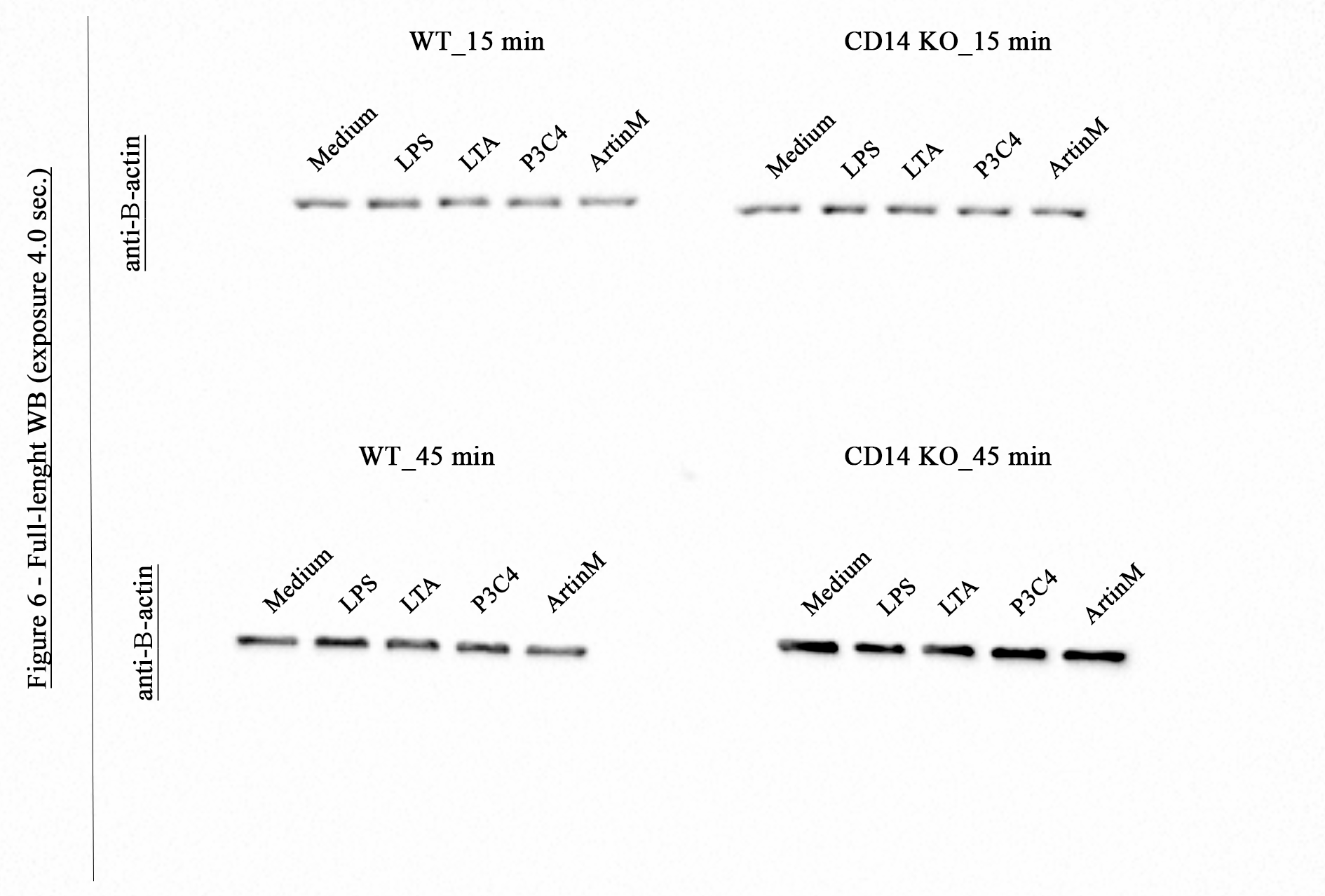


###
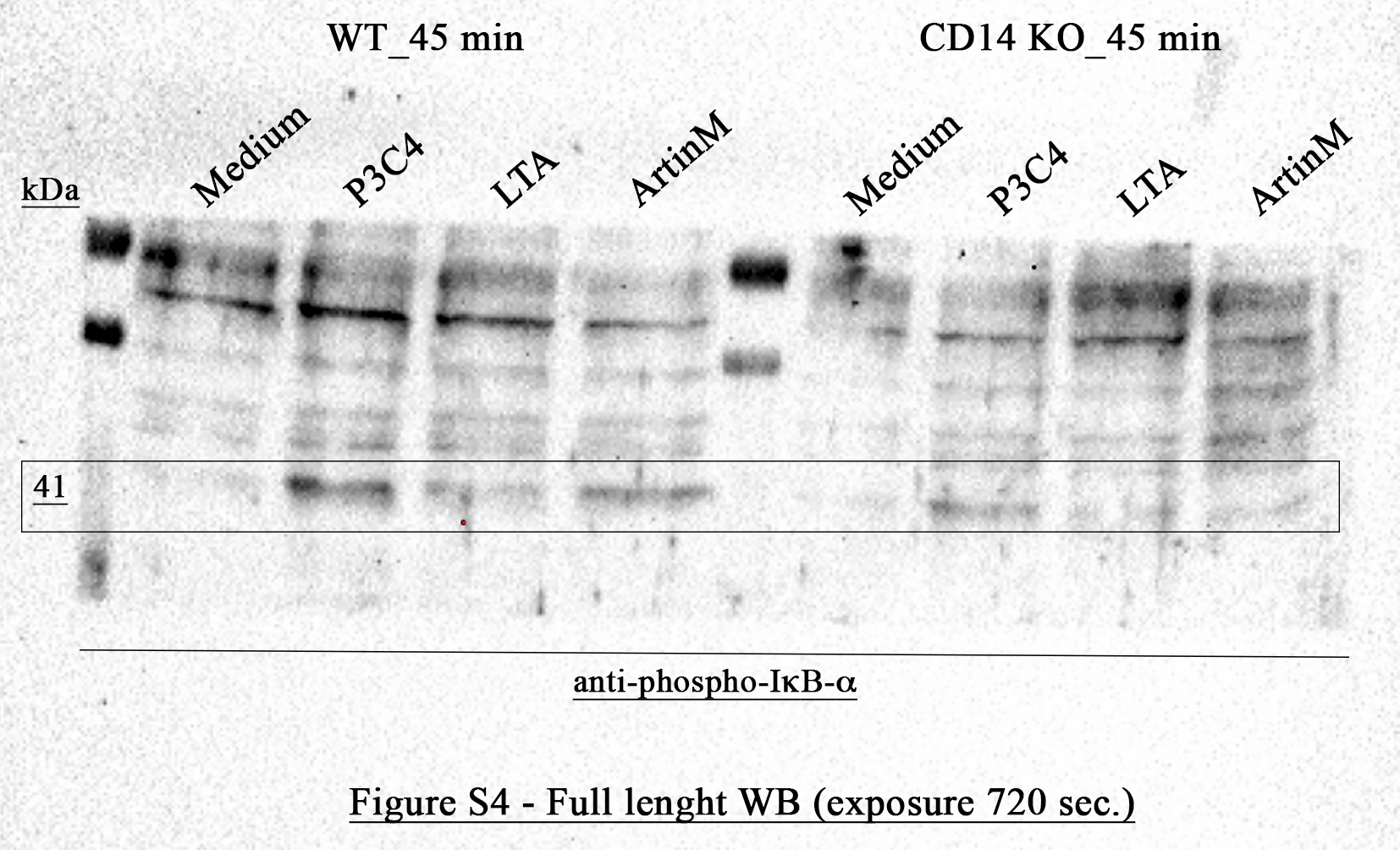


###
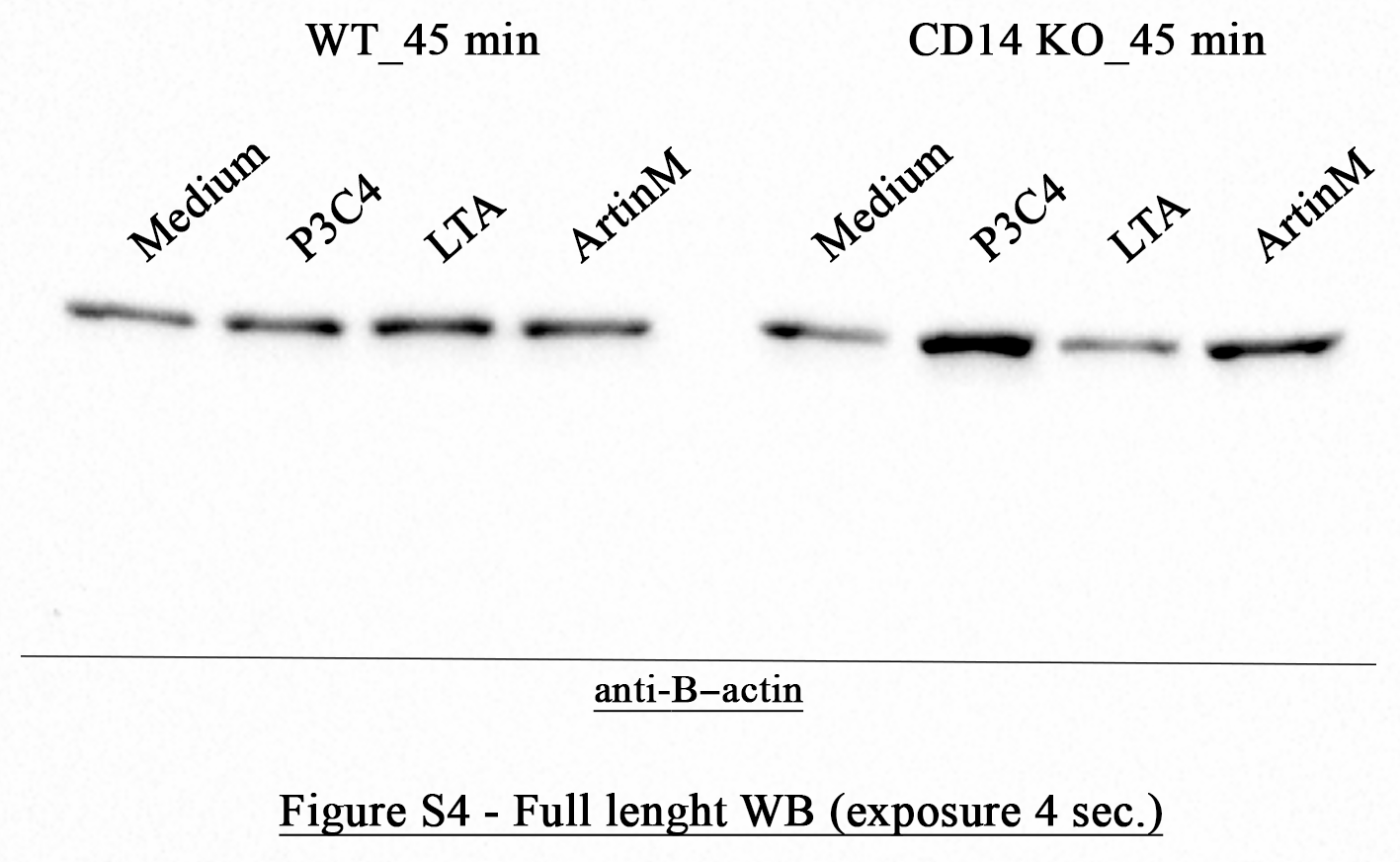

Supplement: Supplementary file 1 — Supplementary Information [file 41598_2017_7397_MOESM1_ESM.doc]
